# A Rationale for the Use of Clotted Vertebral Bone Marrow to Aid Tissue Regeneration Following Spinal Surgery

**DOI:** 10.1038/s41598-020-60934-2

**Published:** 2020-03-05

**Authors:** F. Salamanna, D. Contartese, G. Giavaresi, L. Sicuro, G. Barbanti Brodano, A. Gasbarrini, M. Fini

**Affiliations:** 1 Laboratory of Preclinical and Surgical Studies, IRCCS Istituto Ortopedico Rizzoli, Bologna, Italy; 2 Department of Oncological and Degenerative Spine Surgery, IRCCS Istituto Ortopedico Rizzoli, Bologna, Italy

**Keywords:** Cell biology, Diseases

## Abstract

Vertebral body bone marrow aspirate (V-BMA), easily accessible simultaneously with the preparation of the site for pedicle screw insertion during spinal procedures, is becoming an increasingly used cell therapy approach in spinal surgery. However, the main drawbacks for V-BMA use are the lack of a standardized procedure and of a structural texture with the possibility of diffusion away from the implant site. The aim of this study was to evaluate, characterize and compare the biological characteristics of MSCs from clotted V-BMA and MSCs from whole and concentrate V-BMAs. MSCs from clotted V-BMA showed the highest cell viability and growth factors expression (TGF-β, VEGF-A, FGF2), the greatest colony forming unit (CFU) potency, cellular homogeneity, ability to differentiate towards the osteogenic (COL1AI, TNFRSF11B, BGLAP) and chondrogenic phenotype (SOX9) and the lowest ability to differentiate toward the adipogenic lineage (ADIPOQ) in comparison to all the other culture conditions. Additionally, results revealed that MSCs, differently isolated, expressed different level of HOX and TALE signatures and that PBX1 and MEIS3 were down-regulated in MSCs from clotted V-BMA in comparison to concentrated one. The study demonstrated for the first time that the cellular source inside the clotted V-BMA showed the best biological properties, representing an alternative and advanced cell therapy approach for patients undergoing spinal surgery.

## Introduction

Bone marrow aspiration is an easy, safe and inexpensive method that makes possible a direct transplantation of mesenchymal stem cells (MSCs), endothelial progenitor cells, hematopoietic stem cells, other progenitor cells, growth factors, e.g. bone morphogenetic proteins (BMPs), platelet-derived growth factor (PDGF), transforming growth factor-β (TGF-β), vascular endothelial growth factor (VEGF), and several interleukins, into the defect site^1–3^. Despite only a very small percentage (0.01–0.001%) of MSCs is found among the totality of mononuclear cells in bone marrow aspirate (BMA), the attendance of non-adherent osteogenic cells and the potential collaboration among BMA cell types in tissue repair suggest that the use of whole BMA, instead of expanded, concentrated and purified MSCs, is preferable for bone cell therapy^4^. Recently the use of iliac crest BMA is becoming increasingly trendy also in spinal surgery in order to overcome the problems linked to the harvest and use of iliac crest autograft and/or local autograft (spinous processes, laminae)^5,6^. However, several issues of the process need specific attention in order to optimize the efficacy and reduce potential side effects^7^. In addition, although bone marrow is commonly aspirated by the iliac crest, during spinal surgery its harvest leads to an increase in operative and rehabilitation time and to further morbidity in the donor site^8^. These complications are particularly critical in adolescent idiopathic scoliosis patients who have no pain before surgery and in osteoporotic patients that have a greatest risk of bleeding complications, involuntary penetration of the inner cortex of the ilium and nerve injury^9–11^. To overcome these limitations, several studies explored different BMA harvest sites, finding that the vertebral body, accessible by transpedicular aspiration during spinal procedures, is a robust site for aspiration^12–14^. Additionally we recently demonstrated that human MSCs from whole vertebral body BMA (V-BMA) (not concentrated and/or purified) were successfully isolated and showed best biological properties^14^. It was also shown that MSCs from whole V-BMA expressed definite level of HOX genes, genes able to control characteristic morphologies along regions of the vertebral column, and that HOXB8, a gene with key role in the regulation of adult MSCs, was up-regulated with greater efficiency in comparison to MSCs obtained from concentrated V-BMA^14^. Given the easy availability, safety and biological performances offered by V- BMA use for spinal fusion procedures, we have thought to evaluate clotted V-BMA. This choice started from a previous systematic literature review where the potential use of clotted BMA emerged as a novel cell therapy strategy for orthopedic procedures^15^. This review highlighted that, although there are very few studies about clotted BMA, and most of them are on cartilage regeneration (i.e. microfractures), BMA clot seems to be an effective, reliable, and easy strategy favoring tissue regeneration. A recent preclinical *in vivo* study carried out by Lim *et al*. demonstrated that clotted BMA from iliac crest alone can be considered as an alternative option to autograft for long bone healing^16^. Thus, theoretically, these data demonstrated that clotted BMA represent an attractive approach for bone regeneration. These promising results were probably due to the intricate cascade of chemical reactions during the process of coagulation that comprise platelets degranulation, which allows the release of several osteotropic cytokines and growth factors^17,18^. Additionally, it is necessary to emphasize that the fibrinolytic activity that takes place during the first days within a clot could offer a further source of angiogenic factors (fibrin split products)^17^. However, to date, no preclinical study assessed the *in vitro* biological properties and characteristics of MSCs derived from clotted BMA and evaluated the potential advantages of this alternative and little known approach to the current standard techniques.

By using human V-BMA, the main objective of this study was to evaluate, characterize and compare the biologics characteristics of MSCs from clotted V-BMA to MSCs from whole and concentrate V-BMAs. Using different qualitative and quantitative analyses, we want to prove if clotted V-BMA could be effectively used to enhance spinal fusion procedures offering significant advantages over whole and concentrate V-BMA, providing a novel, advanced and alternative strategy favoring tissue regeneration.

## Results

### Cell morphology and viability

**Figure 1:**
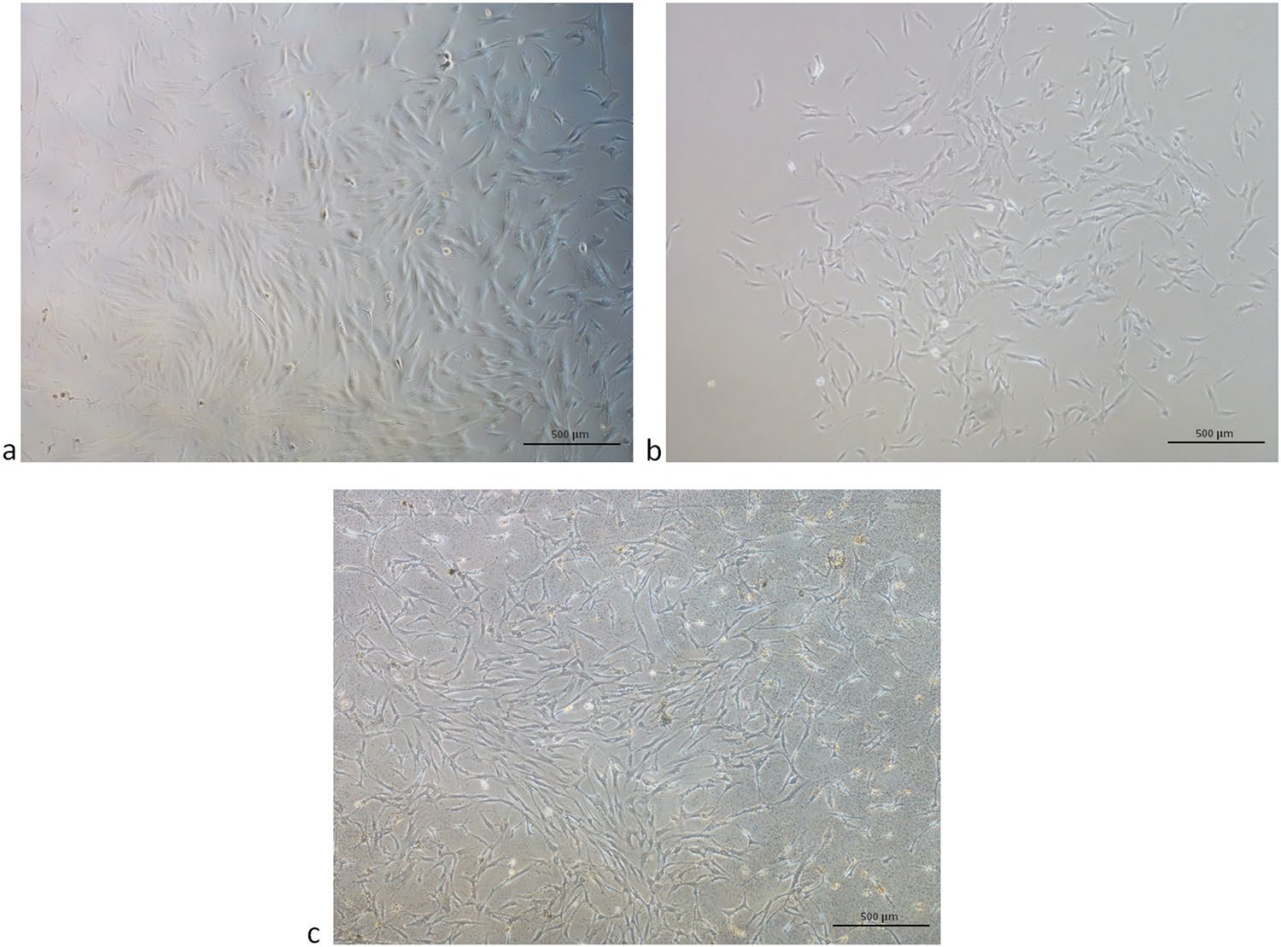
Cells morphology after 10 days of culture of MSCs from (**a**) whole BMA, (**b**) concentrated BMA and (**c**) clotted BMA. Magnification 4×.

In MSCs derived from whole and clotted V-BMAs red blood cells, platelets, and leucocytes were still observable within the first week of culture. However, after 10 days of culture most of the impure cells were discarded and at 14 days a homogenous population of spindle-shaped and plastic-adherent cells was observable in all culture conditions (Fig. 1).

**Figure 2:**
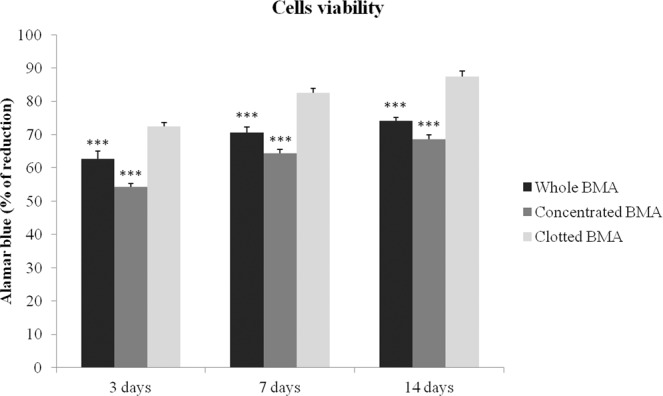
Cell viability by Alamar blue dye performed on MSCs derived from whole BMA (dark bar), concentrated BMA (grey bar) and clotted BMA (light bar) after 3, 7 and 14 days of culture (***p < 0.0005) in basal medium. 3, 7 and 14 days: ***Whole and clotted BMAs *vs*. concentrated BMA; ***clotted BMA *vs*. whole BMA.

Three, 7 and 14 days after cells culture, MSCs from whole and clotted V-BMA showed significantly higher viability in comparison to MSCs from concentrated V-BMA (***p < 0.0005) (Fig. 2). Significantly higher values of cells viability were found at all experimental time in MSCs from clotted V-BMA also in comparison to MSCs from whole V-BMA (***p < 0.0005) (Fig. 2).

### Expression of surface markers

In all culture conditions MSCs were almost completely negative for CD45 (leukocyte common antigen) and CD34 (gp105–120), haematopoietic surface markers, and for CD31 (platelet endothelial cell adhesion molecule) which indicated that they were not of endothelial origin. MSCs in all culture conditions expressed ecto-5′-nucleotidase (CD73), CD90 (thymocyte differentiation antigen-1, Thy-1) and matrix receptor CD105 (endoglin, SH2). However, the isolated cells highlighted a distinct phenotypic population (>90% homogeneous) only in clotted V-BMA. In fact, a percentage <90 were found in MSCs from whole V-BMA for CD73 and in MSCs from concentrated V-BMA for CD44, CD73 and CD90 (Table 1).

**Table 1:** Immunophenotypic profile of MSCs derived from whole, concentrated and clotted BMAs analyzed by flow cytometry (n = 6).

	Whole BMA	Concentrated BMA	Clotted BMA
CD31	2%	1%	10%
CD45	1%	0%	1%
CD34	1%	1%	10%
CD44	93%	52%	100%
CD73	84%	64%	100%
CD90	96%	87%	99%
CD105	98%	97%	95%

### Colony-forming units assay

**Figure 3:**
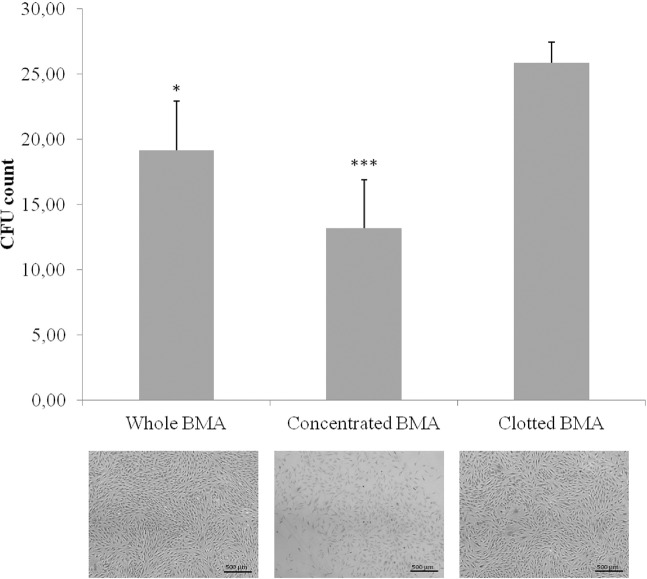
Colony forming units (CFUs) count of MSCs derived from whole, concentrated and clotted BMAs after 10 days of culture in basal medium. The graphs indicate the number of positive colonies/well by toluidine blue staining. *Clotted BMA *vs*. whole BMA; ***Clotted BMA *vs*. concentrated BMA. *p<0.05; ***p < 0.0005. Representative images of CFUs observed by toluidine blue staining after10 days of culture, magnification 4×.

CFUs were significantly increased in MSCs from clotted V-BMA (25.83 ± 1.60) in comparison to MSCs from whole (19.33 ± 3.79: p < 0.05) and concentrated V-BMAs (13.33 ± 3.79; *p* < 0.0005) (Fig. 3).

### *In vitro* osteogenic, adipogenic and chondrogenic differentiation

**Figure 4:**
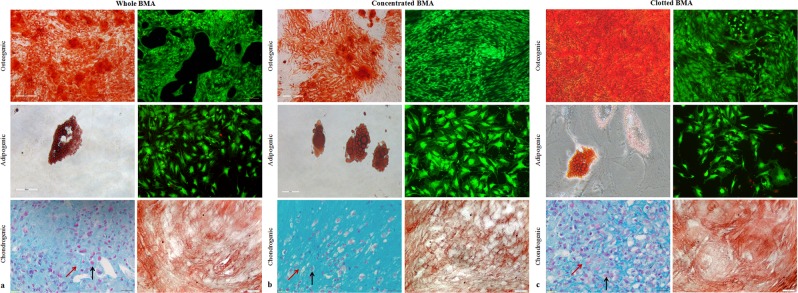
Representative images of *in vitro* osteogenic (Left: Alizarin Red S staining, magnification 4×; Right: LIVE/DEAD fluorescent staining, magnification 10×), adipogenic (Left: Oil Red O, magnification 40×; Right: LIVE/DEAD fluorescent staining, magnification 10×) and chondrogenic (Left: Alcian Blue/Nuclear Fast Red staining, magnification 80×; Right: Collagen Type II magnification 80×) differentiation of MSCs from (**a**) whole, (**b**) concentrated and (**c**) clotted BMAs. Black arrows: chodrocytes; Red arrows: extracellular matrix.

In all culture conditions, LIVE/DEAD staining confirmed the viability of MSCs exposed to osteogenic and adipogenic medium (Fig. 4). Osteogenic induced-MSCs derived from whole, concentrated and clotted V-BMAs showed a change of cells shape and presence of mineralized matrix (red staining). However, in MSCs from whole and clotted V-BMAs a greater amount of calcium deposits were seen and this aspect was more marked in clotted V-BMA (Fig. 4). A decrease in cell density and a change of cells shape, that become more elongated and flat, were observed in all culture conditions when MSCs were exposed to adipogenic medium. The reduction in cell density was more evident in particular for MSCs from whole and clotted V-BMAs (Fig. 4). Concerning the chondrogenic differentiation of MSCs, in all culture conditions the presence of chondrocytes inside the lacunae separated by extracellular matrix was seen (Fig. 4). Although in all culture conditions MSCs differentiated towards the chondrogenic phenotype, MSCs from clotted V-BMA highlighted a greater amount of chondrocytes that were also organized in isogenous group (Fig. 4). Immunostaining showed the presence of Collagen Type II in all MSCs micromasses with more prominent staining in MSCs derived from whole and clotted V-BMAs, while the staining was less evident in MSCs derived from concentrated V-BMA (Fig. 4).

### Gene expression analysis

**Figure 5:**
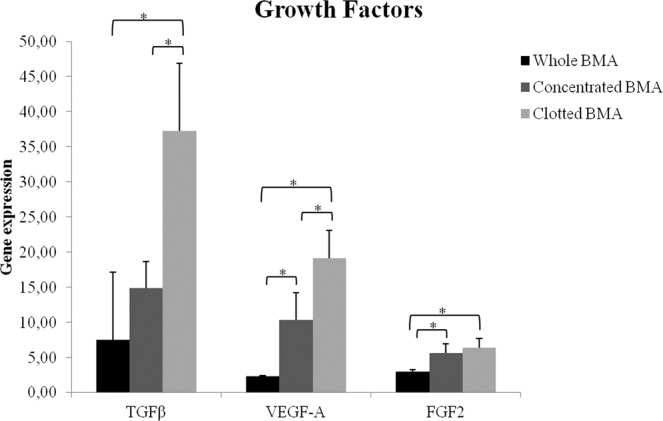
Gene expression measured by quantitative reverse transcriptase polymerase chain reaction of TGF-β, VEGF and EGF in MSCs from whole, concentrated and clotted BMAs. TGF-β, VEGF-A: *MSCs from clotted V-BMA *vs*. MSCs from concentrated V-BMA and MSCs from whole V-BMA; EGF2: *MSCs from clotted V-BMA *vs*. MSCs from whole V-BMA; VEGF-A, FGF2: *MSCs from concentrated V-BMA *vs*. MSCs from whole V-BMA. *p < 0.05.

TGF-β and VEGF-A gene expression showed significant higher values in MSCs from clotted V-BMA in comparison to MSCs from concentrated V-BMA (TGF-β: *p* < 0.05; VEGF: *p* < 0.05) (Fig. 5). Additionally, MSCs from clotted V-BMA revealed significantly higher values of TGF-β, VEGF-A and also FGF2 in comparison to MSCs from whole V-BMA (*p* < 0.05) (Fig. 5). Finally, significantly higher values were also seen for VEGF-A and FGF2 in MSCs from concentrated V-BMA in comparison to MSCs from whole V-BMA (*p* < 0.05) (Fig. 5).

**Figure 6:**
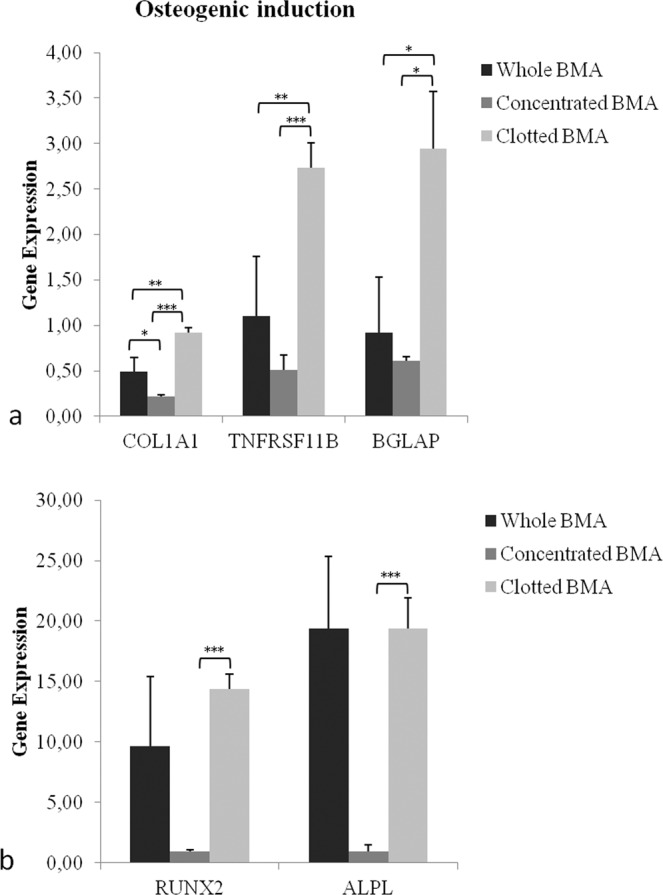
Gene expression measured by quantitative reverse transcriptase polymerase chain reaction of osteogenic markers comparing the differentiation potential of MSCs from whole BMA, concentrated BMA, clotted BMA after 21 days of culture. (**a**) COL1A1: ***clotted BMA *vs*. concentrated BMA; **clotted BMA *vs*. whole BMA; *whole BMA *vs*. concentrated BMA. TNFRSF11B: ***clotted BMA *vs*. concentrated BMA; **clotted BMA *vs*. whole BMA. BGLAP: *clotted BMA *vs*. concentrated BMA; *clotted BMA *vs*. whole BMA. RUNX2: ***clotted BMA *vs*. concentrated BMA. ALP: ***clotted BMA *vs*. concentrated BMA. *p < 0.05; **p < 0.005; ***p < 0.0005.

After 21 days of osteogenic induction, RT-PCR demonstrated that MSCs from clotted V-BMA showed significantly higher expression of COL1A1, TNFRSF11B and BGLAP in comparison to MSCs from concentrated (COL1A1: *p* < 0.0005; TNFRSF11B: *p* < 0.0005; BGLAP: *p* < 0.05) and whole V-BMAs (COL1A1: *p* < 0.005; TNFRSF11B: *p* < 0.005; BGLAP: *p* < 0.05) (Fig. 6a). Likewise, significantly higher values of RUNX2 and ALP were seen for clotted V-BMA in comparison to concentrate ones (RUNX2 and ALP: *p* < 0.0005) (Fig. 6b). Finally, COL1A1 (*p* < 0.05) also had significantly higher values in whole V-BMA when compared to concentrated V-BMA (Fig. 6a).

**Figure 7:**
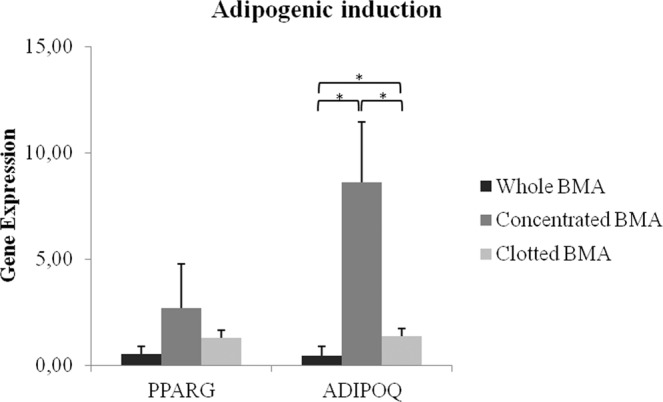
Gene expression measured by quantitative reverse transcriptase polymerase chain reaction of adipogenic markers comparing the differentiation potential of MSCs from whole BMA, concentrated BMA, clotted BMA after 21 days of culture. ADIPOQ: *concentrated BMA *vs*. whole BMA and clotted BMA; *whole BMA *vs*. clotted BMA.*p < 0.05.

Adipogenic differentiation of MSCs from whole and clotted V-BMAs was significantly reduced (*p* < 0.05) in comparison to MSCs from concentrated V-BMA, as shown by ADIPOQ expression (Fig. 7). Additionally, MSCs from whole V-BMA had a significant lower value of ADIPOQ (*p* < 0.05) in comparison to MSCs from clotted B V-MA (Fig. 7).

**Figure 8:**
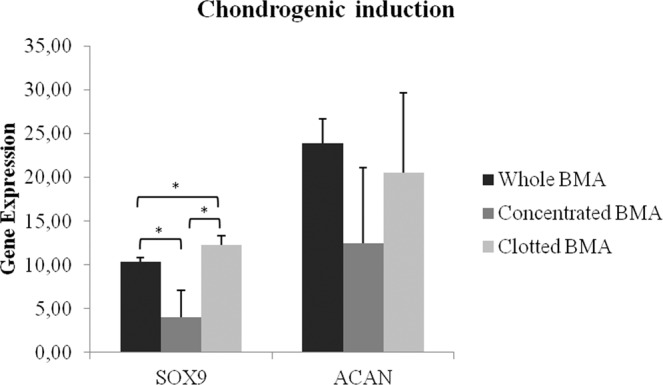
Gene expression measured by quantitative reverse transcriptase polymerase chain reaction of chondrogenic markers comparing the differentiation potential of MSCs from whole BMA, concentrated BMA, clotted BMA after 30 days of culture. SOX9: *clotted BMA *vs*. whole and concentrated BMA; *whole BMA *vs*. concentrated BMA. *p < 0.05.

Chondrogenic differentiation capability revealed a significant higher value of SOX9 expression (*p* < 0.05) in clotted V-BMA in comparison to whole and concentrated V-BMAs (Fig. 8). Significant differences were also seen for MSCs derived from whole V-BMA that highlighted a higher value of SOX9 in comparison to MSCs from concentrated V-BMA (*p* < 0.05) (Fig. 8).

**Figure 9:**
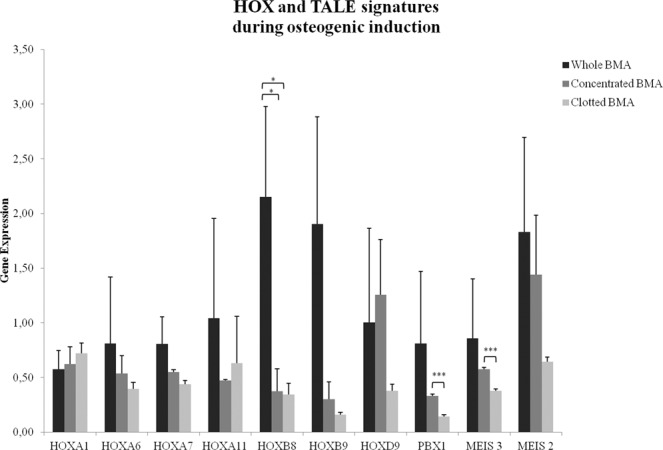
Gene expression measured by quantitative reverse transcriptase polymerase chain reaction of HOX and TALE signatures for MSCs from whole BMA, concentrated BMA, clotted BMA after 21 days of osteogenic induction. HOXB8: *whole BMA *vs*. concentrated BMA and clotted BMA; PBX1 and MEIS3: ***concentrated BMA *vs*. clotted BMA. *p < 0.05; ***p < 0.0005.

After 21 days of osteogenic induction PBX1 and MEIS3 (*p* < 0.0005) showed significantly higher values in MSCs from concentrated V-BMA in comparison to MSCs from clotted V-BMA (Fig. 9). Additionally, significantly higher values (*p* < 0.05) were found also for HOXB8 in MSCs from whole V-BMA in comparison to MSCs isolated from concentrated and clotted V-BMAs (Fig. 9).

## Discussion

**Figure 10:**
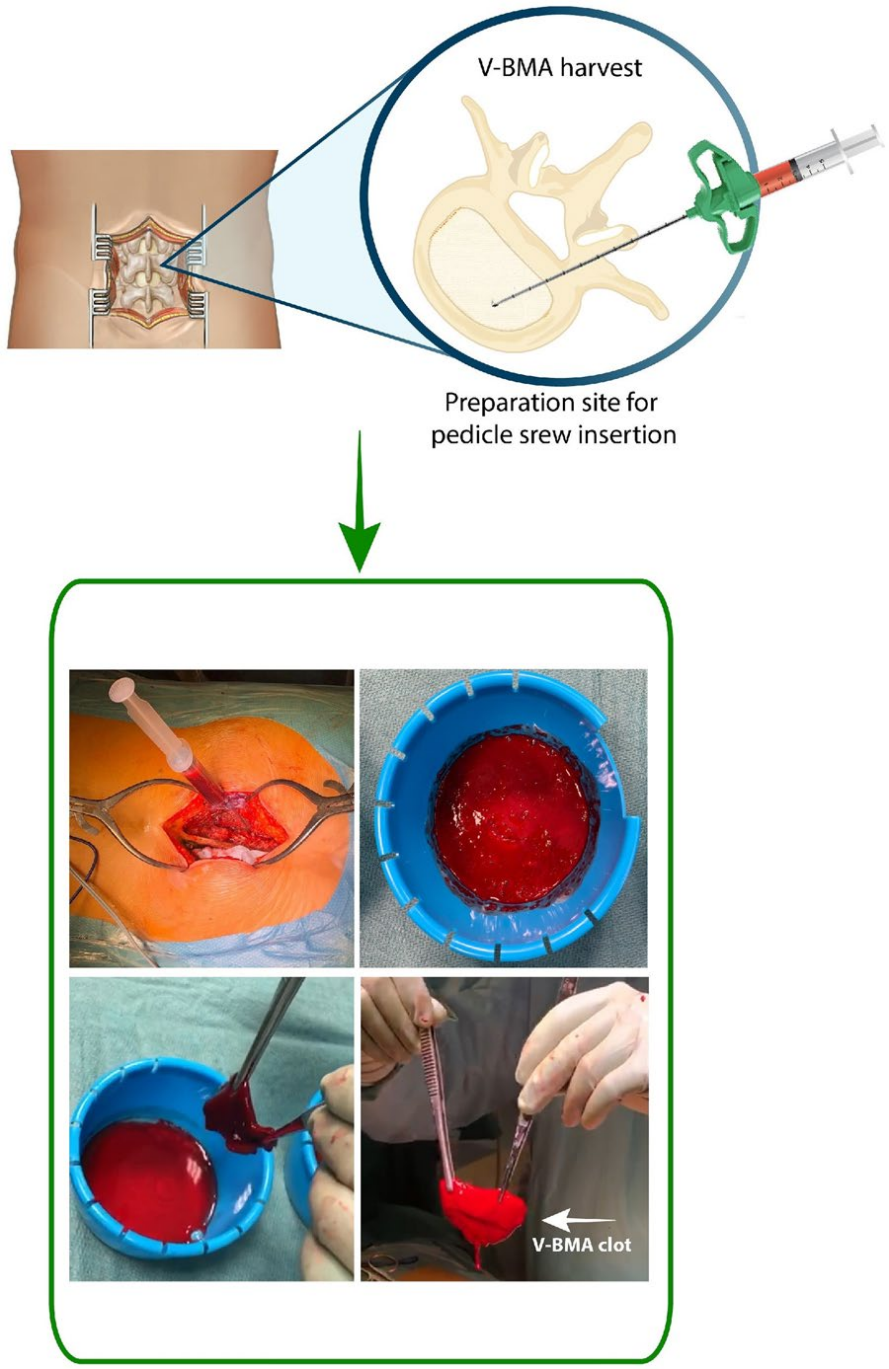
Surgical feasibility of V-BMA clot procedure in the clinical theatre.

Spine fusion success depends on different factors, several linked to the patient (age, smoking, previous spine surgery, osteoporosis, osteoarthritis) and other linked to the surgery, such as use of instrumentation, surgical approach, use of bone graft/substitutes^19,20^. The increase of aging population and the need for multilevel spinal fusion surgery, request techniques capable to increase success rate of fusion and reduce pseudarthrosis rate^21^. A promising approach is the use of BMA from iliac crest^6,21^. Despite the clinical favorable outcome, its use is still limited due to several complications, such as increase in operative and rehabilitation time and to additional morbidity in the donor site^22^. To overcome these limitations V-BMA, accessible by transpedicular aspiration during spinal procedures, represent a promising cell source for fusion and its minimally invasive application may represent an efficacious biological cell source for spinal surgery outcomes improvement^12,14^. In this study we propose to evaluate and compare MSCs from clotted V-BMA as advanced and alternative cell therapy approach, with best biological properties and characteristics than MSCs derived from whole and concentrated V-BMA, for spinal fusion surgery. We hypothesized that despite V-BMA clot formation is usually considered a complication hampering its use during spinal surgery its biological and physical properties could make it a good candidate as autologous cell therapy in spinal surgery. The V-BMA clot with its complex environment of many cell types, growth factors and osteotropic cytokines is supposed to perform the necessary physiological functions to accelerate spinal fusion. Thanks to its structural texture V-BMA clot could be also placed where necessary at the implant site maximizing efficacy and minimizing clinical complications. However, main drawbacks in the use of clotted V-BMA could be the real surgical feasibility of the procedure in the clinical theatre and the need to have high amounts of V-BMA in presence of multilevel (long) spinal fusion. To confirm the surgical feasibility of this procedure we tested, directly in the clinical theatre, for the first time, the technical feasibility (Fig. 10) demonstrating its practicability. Concerning the need of high amounts of V-BMA in presence of multilevel (long) spinal fusion we assume that begin able to harvest V-BMA from each site for pedicle screw insertion there will be no problem to obtain the required amount of V-BMA. Finally, this easier procedure could totally avoid the burden of the bench to bedside translation of the use of this strategy also increasing the cost-effectiveness of the intervention.

Our results showed that at all experimental time *in vitro* cells viability and CFU potency of MSCs from clotted V-BMA were increased compared to all the other culture conditions. These results may be due to the culture methods, i.e. to the clot by itself, that initially presents erythrocytes and platelets but also to the presence of a greater amount of growth factors, i.e. TGF-β, VEGF-A and FGF2, issued by the α-granules of platelets during the coagulation of bone marrow^23–25^. This clinically relevant source of growth factors present in greater amount in the clotted V-BMA may be more effective in the clinical *scenario* where increased vascularity and healing are mandatory. Additionally, TGF-β is reported to have anabolic and anti-inflammatory effects and it is known for its regulation of MSC proliferation and colonies formation^26^. VEGF, which is part of a subset of the PGDF family, is as a potent promoter of angiogenesis and has a powerful role in tissue healing^27^. Concerning FGF2, it was seen that it enhances MSCs proliferation and cooperates in maintaining MSCs three lineage differentiation potential during *in vitro* expansion^28^.

With reference to cellular homogeneity, it was seen that in V-BMA clot about 10% of cells were positive for CD31 and CD34 markers, thus suggesting that these cells may contain several populations with different phenotypic and biological properties. However, despite the currently adopted MSC definition relies on the International Society for Cellular Therapy criteria (adherence to plastic under standard culture conditions, expression or lack of expression specific surface markers, *in-vitro* three-lineage differentiation potential), functional and phenotypic differences may exist across tissue sources, culture conditions, and extent of *ex-vivo* expansion^29,30^. In fact, it is important to emphasize that although prevailing opinion states that MSC are CD34−, *in vitro* characterization mainly occurs following some passages of culture. This aspect not represents the *in vivo* or originally extracted cell phenotype. In fact, freshly extracted stem cells, from different anatomical sites, have shown to hold CD34+ cells^31–33^. CD34+ MSC are there immediately following withdrawal but quickly diminish after a short time in culture^34,35^. In addition, CD34 expression was linked with long-term proliferative capability and elevated colony forming efficiency^34,36^. CD34 has also been found on embryonic stem cell derived MSC, thus suggesting to be a marker of premature human MSC^33,37^. Also concerning CD31, greatly expressed on endothelial cells and to diverse degrees also on some hematopoietic cells, including monocytes, granulocytes and platelet^38^, it was reported the existence of large multinuclear CD31+ cells in the beginning of human bone marrow MSCs culture^39^. These observations imply that MSCs from V-BMA clot ‘heterogeneity’ is not an isolated case; however, this aspect must be further investigated to determine whether co-expression of specific MSC markers confers to V-BMA clot cells specific and peculiar properties. Additionally, differently, for the other culture condition, and in particular for concentrated V-BMA, a decrease in MSCs population, positive for CD44 and CD73, were also observed. This aspect is of critical importance because it can lead to different degrees of effectiveness after MSCs transplantation^40^. CD44 that was expressed by 93% of MSCs from whole V-BMA, 52% of MSCs from concentrated V-BMA and 100% of MSCs from clotted V-BMA regulates and operates in cell adhesion, migration, homing, proliferation and apoptosis and have also a role in stemness properties and maintenance^41,42^. CD73+ positive populations that in our study were expressed only by 64% of MSCs from concentrated V-BMA and by high percentage in the other culture conditions are significantly enriched in clonogenic cells, able to generate CFUs^43^. In our study, this data was confirmed also by the lower CFUs number that was evaluated in MSCs from concentrated V-BMA. Concerning CD90, that was expressed by high percentage in MSCs from whole and clotted V-BMAs and by a lower percentage in MSCs from concentrate V-BMA, it has a critical role in stem cell growth and differentiation potential^44^. In fact, even if all culture conditions produce mature cells of all mesenchymal lineages when differentiating into osteocytes, adipocytes and chondrocytes, RT-PCR revealed different regulation of genes marker for the three-lineage differentiation between the examined methods of isolation. A significant increase of genes marker of osteogenic differentiation, i.e. COL1AI, TNFRSF11B and BGLAP, were observed in MSCs from clotted V-BMA in comparison to all culture conditions. The COL1AI down-regulation in MSCs from whole and concentrate V-BMAs underlines a reduction in their final osteogenic potential^45^. In our study a down-regulation was also observed for TNFRSF11B (osteoprotegerin) expression, a member of tumor necrosis factor receptor super family, principally produced by osteoblasts and osteocytes, able to reduce osteoclasts activities^46^. Similarly, gene expression levels of osteocalcin (BGLAP), a protein released by adult osteoblasts, was down-regulated in MSCs from whole and concentrate V-BMA^47^. MSCs from clotted V-BMA showed also higher expression level of RUNX2, the key inducer of ALPL, COL1A1 and BGLAP genes, and ALPL in comparison to MSCs from concentrated V-BMA. These results additionally confirmed the data obtained by Alizarin Red S staining that showed a greater presence of calcium phosphate deposits in MSCs from clotted V-BMA, with a more intense red-orange staining of mineralized bone matrix. The higher incidence of calcium phosphate deposits and the higher expression of osteogenic markers in MSCs from clotted V-BMA proved the greater bone formation ability of these cells. Similarly to the osteogenic differentiation also the chondrogenic differentiation ability was higher in MSCs from clotted V-BMA, as confirmed by Collagen Type II immunostaining and SOX9 gene expression, a member of the family of Sox (Sry-type HMG box) genes. SOX9, defined as a “master regulator” of the chondrocyte phenotype, is critical for the chondrogenic differentiation ability and for the production of extracellular matrix^48^. Unlike osteogenic and chondrogenic differentiation ability, adipogenic one showed few lipid vacuoles (Oil Red staining) and lower ADIPOQ gene expression in MSCs from clotted V-BMA. These results indicated that MSCs from clotted V-BMA were able to differentiate onto the adipogenic lineage when exposed to definite inducing factors but with a lower ability in comparison with all the other culture condition. This greater ability to differentiate towards the osteogenic and chondrogenic phenotype associated to the lower ability to differentiate toward the adipogenic lineage, provide to the MSCs isolated from clotted V-BMA the best biological properties and characteristics.

We recently demonstrated that vertebral MSCs, isolated through different methods, expressed diverse level of HOX and TALE signatures, genes that control specific vertebral elements morphology. Thus, also in this study we analyzed whether MSCs from whole, concentrated and clotted vertebral V-BMAs might, after osteogenic induction, modulate the expression of these genes^14,49^. Our results revealed that MSCs from whole, concentrated and clotted V-BMAs express different level of HOX and TALE genes with significant differences for PBX1 and MEIS3 between MSCs from concentrated and clotted V-BMAs. This last showed reduced level of these genes. Although there are no reports regarding the screening of TALE groups that are involved in the osteogenesis of vertebral MSCs, it was reported that PBX1 is down-regulated during osteoblast maturation since its presence in osteoprogenitor cells and its reduction in mature osteoblasts^50^. These data were also confirmed by the fact that PBX1 depletion by shRNA increases osteoblast differentiation^50^. Additionally, in accordance with our results it was also reported that increased PBX1 level in MSCs reduced the expression of early (RUNX2 and ALPL) and late osteoblast genes (BGLAP), whereas decreased levels PBX1 lead to increased osteoblast gene expression^50^. Concerning MEIS3 expression no specific data were reported concerning its role during MSCs osteogenic differentiation ability. However, Roson-Burgo at al. supposed MEIS3 as interactor of Kruppel-like factor 4 (KLF4) in human bone marrow MSCs^51^. KLF4 showed a possible control over mesenchymal differentiation and it was seen that KLF4 DNA-binding protein presents a critical role in the regulation of MSC transcriptional activity, thus acts in maintaining cells in an undifferentiated state^52,53^. Thus, as for KLF4, we hypothesized also for MEIS3 a down-regulation when vertebral MSCs were submitted to differentiation pressure. Finally, concerning the highest expression level of HOXB8 in MSCs from whole V-BMA this data confirmed our previous study where this gene, involved in the hematopoietic stem and early progenitor cells expansion^54^, was strongly transcribed in whole V-BMA^14^.

Until now, the real biological properties and characteristics of clotted V-BMA are not yet evaluated. Our study demonstrated for the first time that the cellular source inside the vertebral clotted BMA showed the best biological properties, representing an alternative strategy for patients undergoing spinal surgery. The use of vertebral clotted BMA not only eliminates the need to concentrated and/or purified BMA but also gives an attractive tool for cellular therapy able to provide higher biomechanical stability to the graft site in comparison to the existing approaches (i.e. whole and concentrated V-BMAs). Vertebral BMA can be harvested at the same time of the preparation of the site for pedicle screw insertion and reintroduced after clotting (15–30 minutes) in the fusion site alone and/or in combination with a graft, without extra surgical time or involvement of other donor site. We hypothesize that the easier clinical access to vertebral BMA in the patient, the need of cellular *in vitro* expansion/purification and the ability to offer a greater stability to the graft site will totally avoid the burden of the *‘bench to bedside translation’* of the use of this procedure also decreasing the cost-effectiveness of the intervention.

## Materials and Methods

### Bone marrow aspirate collection and processing

**Figure 11:**
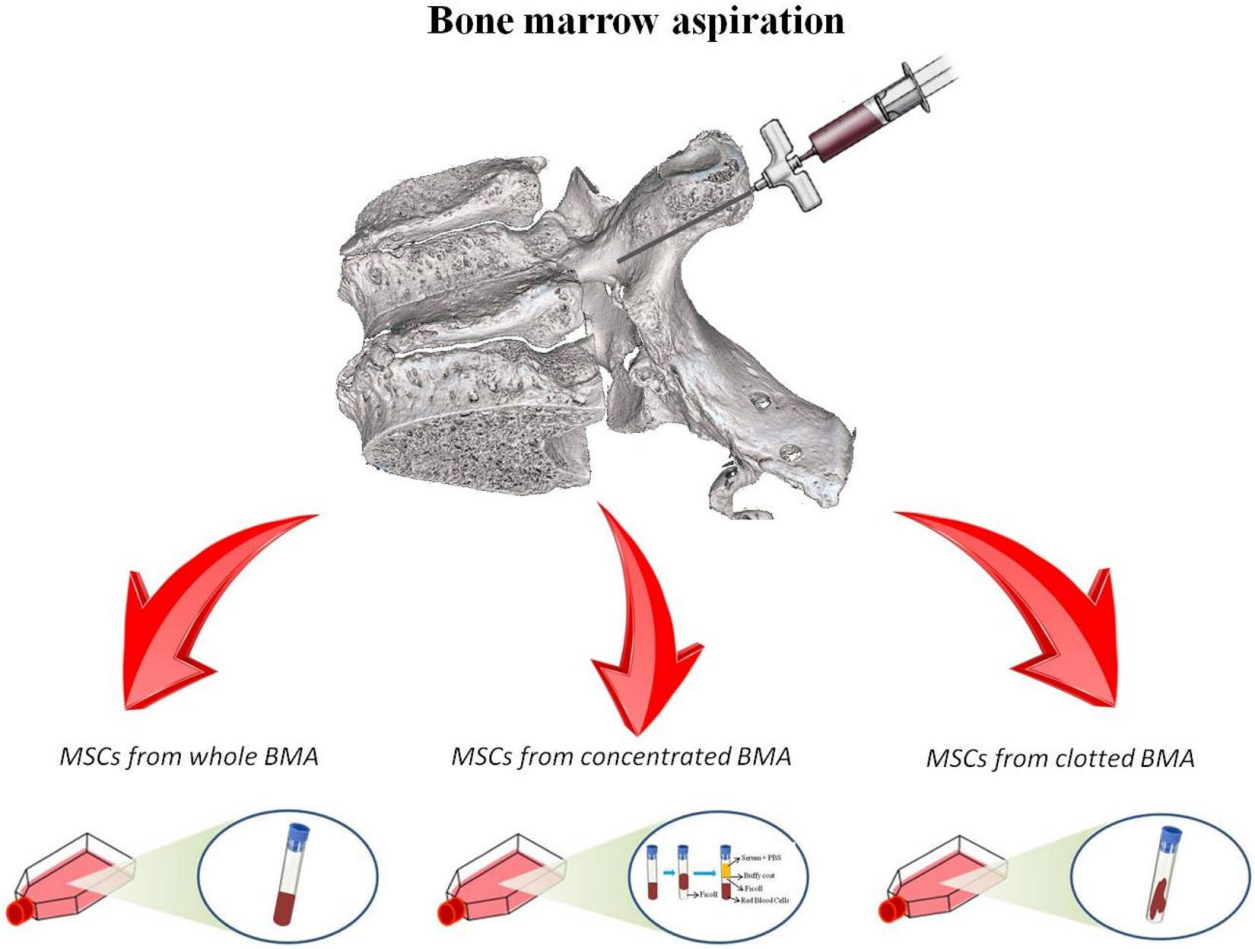
Study experimental set-up. Human BMA harvest from vertebrae divided in 3 equal parts: two parts placed into test tubes containing heparin as anticoagulant for MSCs isolation from whole and concentrated BMAs, one part placed in test tube without any anticoagulant for MSCs isolation from clotted BMA.

The study was approved by the Ethics Committee of IRCCS Istituto Ortopedico Rizzoli (Protocol n. 9499_v-MSC) and was carried out in accordance with relevant guidelines and regulations (IRCCS Istituto Ortopedico Rizzoli has kept the ISO 9001 Quality certification since 2008, with special reference to the Research area). Written informed consent was obtained from all subjects involved in the study. Exclusion criteria were human immunodeficiency virus (HIV), hepatitis B virus (HBV), hepatitis C virus (HCV), diabetes, pregnancy, bone diseases, drugs active on bone metabolism, primary bone tumors, metastases, minors and/or patients incapable of giving consent personally. Four milliliters of human vertebral aspirate was harvested into a 10 ml syringe from each vertebral pedicle, during the preparation of the pilot hole for pedicle screw fixation, of six female patients (mean age: 57.33± 10.19; body mass index and bone mineral index matched) undergoing spinal surgical procedures involving posterolateral arthrodesis. This process was repeated until a total volume of 16 ml bone marrow was obtained from each patient. Vertebral aspirate was divided in 3 equal parts of 5.33 ml each. Two parts have been placed into test tubes containing heparin as an anticoagulant for MSCs isolation from 1) whole V-BMA and 2) concentrated V-BMA. The other part has been placed in test tube without any anticoagulant for MSCs isolation from 3) clotted V-BMA. Subsequently, V-BMAs were transferred to the laboratory and they were processed as described below (Fig. 11).

#### MSCs from whole V-BMA

As previously described^14^, whole V-BMA was immediately put in the culture flask and cultured with Dulbecco’s Modified Eagles Medium (DMEM, Sigma–Aldrich, St. Louis, MO), containing 10% Fetal Bovine Serum (FBS, Lonza), 100 U/ml penicillin, 100 mg/ml streptomycin (Gibco, Life Technologies, Carlsbad, CA) and 5 mg/ml plasmocin (Invivogen, San Diego, CA).

#### MSCs from concentrated V-BMA

V-BMA mononuclear cell fraction was harvested for MSC isolation using conventional density gradient centrifugation (Histopaque, Sigma, Aldrich, MO, USA), as reported in our previous works^14^. Briefly, the entire aspirate volume was mixed 1:1 with phosphate buffer solution (PBS). The diluted bone marrow was subsequently layered on top of Histopaque and centrifuged at 2.200 rpm for 30 minutes. The mononuclear cells in the interface were extracted and washed twice with PBS by centrifuging at 1.800 rpm for 10 minutes. The cells pellet was then suspended and cultured in DMEM containing 10% FBS (Sigma–Aldrich, St. Louis, MO), 100 U/ml penicillin, 100 mg/ml streptomycin (Gibco, Life Technologies, Carlsbad, CA) and 5 mg/ml plasmocin (Invivogen, San Diego, CA).

#### MSCs from clotted V-BMA

V-BMA in the test tube without anticoagulant clotted in about 15–30 minutes and subsequently it was entirely, like an explants, put in a culture flask with Dulbecco’s Modified Eagles Medium (DMEM, Sigma–Aldrich, St. Louis, MO), containing 10% Fetal Bovine Serum (FBS, Lonza), 100 U/ml penicillin, 100 mg/ml streptomycin (Gibco, Life Technologies, Carlsbad, CA) and 5 mg/ml plasmocin (Invivogen, San Diego, CA).

All cultures (whole, concentrated and clotted) were incubated at 37 °C in 5% CO_2_ and under hypoxia (2% O_2_), in order to better mimic the microenvironment of the stem cells niche. Non-adherent cells were washed and culture medium changed after 72 h. Subsequently, culture medium was replaced twice a week, up to 85–90% of cell confluence.

### Cell viability

After removing non-adherent cells (after 3 days), MSCs were observed twice a week up to 85–90% of cell confluence and the images acquired by a standard light microscope (Nikon Eclipse, Ti-U, Nikon Italia Srl, Italy) equipped with a digital camera at 4× and 10× magnification.

At 3, 7 and 14 days of cell culture, Alamar blue dye test (Serotec, Oxford, UK) was used, as previously described^14^, to evaluate cell viability. Briefly, the reagent is a dye, which incorporates an oxidation-reduction indicator that changes color in response to the chemical reduction of growth medium, resulting from cell growth. It was added to each culture well (1:10 v/v) for 4 h at 37 °C. After transferring the supernatants to 96-well plates, the absorbance was read spectrophotometrically at 570- and 600-nm wave lengths (for the fully oxidized and reduced forms of reagent) by MicroPlate reader (BioRad, CA, USA). The results, obtained as optical density (OD), were processed following the manufacturer’s instructions and expressed as reduction percentage.

### Flow cytometry analyses

The characteristics of MSCs from whole, concentrated and clotted V-BMAs were investigated by characterizing cell-surface markers of isolated cells using flow cytometry analysis^12^. To test surface antigen expression, 0.5–1 × 10^5^ MSCs for each antigen were washed with PBS, centrifuged at 260 g for 5 min, and incubated at 4 °C for 30 min in flow cytometry buffer (FCB, 2% FBS in PBS) adding 0.5 μg/ml of fluorescein isothiocyanate (FITC)-conjugated antibody against CD31, CD45, CD34, CD44, CD73, CD90 and CD105. FITC-conjugated nonspecific IgG was used as isotype control (BioLegend, San Diego, CA, USA). Cell fluorescence was evaluated with FACSCanto II instrument (Becton Dickinson, Franklin Lakes, NJ, USA) and analyzed by FACSDiva software (Becton Dickinson).

### Colony-forming unit-fibroblasts (CFU-F) Assay

The number of colony-forming units (CFUs) was evaluated for MSCs from whole, concentrated and clotted V-BMAs. Two-hundred MSCs/cm^2^ were plated onto six-well plates and cultured for 10 days. At the endpoint, cells were fixed with 10% formalin for 20 min and stained with 0.1% toluidine blue in 1% paraformaldehyde (PFA) for 1 h^12,14^. The aggregates with ≥20 cells were visually scored as colonies and counted with a light microscope (Olympus BX51).

### *In vitro* osteogenic, adipogenic and chondrogenic differentiation

Osteogenic, adipogenic, and chondrogenic differentiations were induced for MSCs from whole, concentrated and clotted V-BMAs. To induce osteogenic and adipogenic differentiation, MSCs were plated at a density of 7.0 × 10^3^ cells per cm^2^ onto six-well plates and incubated in culture medium (DMEM with 10% FBS, 100 U/ml penicillin, 100 mg/ml streptomycin and 5 mg/ml plasmocin) for 3 days. After 3 days by initial seeding, the medium was replaced with osteogenic and adipogenic medium and cells were cultured for 21 days. Osteogenic medium consisted of culture medium supplemented with dexamethasone 10^−8^ M (sol.1:100), ascorbic acid 50 μg/ml and β-glycerophospate 10 mM. Adipogenic medium consisted of culture medium supplemented with isobutylmethylxanthine 500 μM, indomethacin 100 μM, dexamethasone 1 × 10^−6^ and insulin 2.5 mg. Osteogenic or adipogenic induction medium was changed every three days for 21 days. LIVE/DEAD staining was used to assess cell viability after 21 days of culture in osteogenic and adipogenic medium. Briefly, LIVE/DEAD staining was performed by incubating the cells with a working solution of calcein AM (4 μm) and ethidium homodimer-1 (1 μm) for 45 minutes at 37 °C. Cells were washed with PBS and images acquired by a fluorescence microscopy (Nikon Eclipse, Ti-U, Nikon Italia Srl, Italy) equipped with a digital camera at 10× and 20× magnification.

To induce chondrogenesis, 2.5 × 10^5^ cells per tube were pelleted (micromasses) at 1.500 rpm for 10 minutes. Pellet cultures were incubated for 3 days in culture medium before being switched to chondrogenic medium and grown for further 30 days. Chondrogenic medium consisted of culture medium supplemented with 5 μg/ml insulin, 5 μg/ml transferrin and 5 μg/ml selenous acid, 0.1 μM dexamethasone, 0.17 mM ascorbic acid–2-phosphate, 1 mM sodium pyruvate, 0.35 mM proline and 10 μg/ml transforming growth factor-β3 (TGF-β3). Chondrogenic induction medium was changed every three days for 30 days^12,14^.

### Staining of differentiated cells

For evaluating differentiation potentials, after 21 days osteogenic cultures were fixed in 10% formaldehyde for 15 minutes and stained with 2% Alizarin Red S (Sigma-Aldrich) for 30 minutes at room temperature to detect calcium depositis. To detect lipid accumulation after 21 days, adipogenic cultures were fixed in 4% paraformaldehyde for 10 minutes at room temperature and stained with 1.8% Oil Red O (Sigma-Aldrich) for 15 minutes at room temperature. After 30 days, chondrogenic pellets (micromasses) were fixed in 10% formaldehyde for 30 min, washed in distilled water, and dehydrated in increasing ethanol series. Finally, they were clarified in xylene (VWR International, Milan, Italy) and embedded in paraffin (Thermo Fisher Scientific, Waltham, MA) blocks. Blocks were sectioned along a transversal plane and cut into 5 μm sections; 3 consecutive sections for each sample were stained with Alcian Blue/Nuclear Fast Red and 2 additional sections were immunostained for Collagen Type II. Briefly, after fixation, sections were rinsed in PBS and permeabilized by incubation in 0.3% hydrogen peroxide in PBS solution for 15 min. Slides were pre-treated for antigen unmasking with 0.2% Pronase (Sigma–Aldrich, US-MO) solution in PBS for 30 minutes at 37 °C. After washing, the slides were incubated at room temperature for 1 hours with Blocking Serum (Vectastain Universal-Quick-Kit, Vectors Laboratories, US-CA) to prevent nonspecific bindings, followed by incubation with specific rabbit polyclonal antibodies against Collagen Type II (NSJ Bioreagents, CA) overnight at 4 °C. After rinsing in PBS, the slides were incubated with an anti-rabbit HRP-conjugated secondary antibody (Bethyl Laboratories, US-TX) and Streptavidin/Peroxidase complex (Vectastain Universal-Quick-Kit). Finally, reactions were developed using Vector NovaRed Substrate Kit for Peroxidase (Vectors Laboratories, US-CA). Negative controls, by omitting the primary antibody, were included to check proper specificity and performance of the applied reagents.

Images were taken using a standard light microscope (Nikon Eclipse, Ti-U, Nikon Italia Srl, Italy) equipped with a digital camera at 4x, 10x, 20x and 80x of magnification.

### RNA isolation and cDNA synthesis

Before three lineage differentiation and at 21 days of osteogenic and adipogenic differentiation, total RNA was isolated by PureLink RNA Mini Kit (Life Technologies, Carlsbad, CA, USA) according to manufacturer’s instructions. For condrogenic differentiation, after 30 days, micromasses were homogenized and total RNA extraction was performed by phenol/chlorophorm method using TRIzol reagent (Invitrogen, Life Technologies, Carlsbad, CA) and chloroform 99% (Sigma-Aldrich); samples were vortexed thoroughly, incubated for 2 min at RT and centrifuged at 12,000 g for 20 min at 4 °C. The resulting aqueous phase was then purified with PureLink RNA mini kit columns (Life Technologies) following manufacturer’s direction. For all samples total RNA was eluted with RNase-free water and quantified by NanoDrop 2000 (Thermo Scientific, Waltham, MA, USA). Purified RNA was reverse transcribed with Superscript VILO cDNA Synthesis kit (Invitrogen, Life Technologies, Carlsbad, CA, USA), reaction was carried out in a 2720 Thermal Cycler (Applied Biosystems, Foster City, CA) at 25 °C for 10 min, 42 °C for 60 min and 85 °C for 5 min. cDNA was diluted for gene expression analysis a concentration of 5 ng/ul with nuclease-free H_2_O^14^.

### Quantitative real-time PCR (qPCR)

Quantitative real-time PCR (qPCR) analysis was performed in a LightCycler Instrument (Roche Diagnostics) using QuantiTect SYBR Green PCR kit (Qiagen, Hilden, Germany). The following protocol was pursued: initial denaturation at 94 °C for 15 min and 40 cycles of amplification (15 s at 94 °C, 20 s at the appropriate annealing temperature for each target and 20 s at 72 °C). The protocol was concluded by melting curve analysis to check for amplicon specificity. The threshold cycles (Ct) were determined for each sample and these values were used for comparative gene expression analysis. For TGF-β, VEGF-A and FGF2 expression it was employed the 2−ΔCt method while for all the others genes we employed 2−ΔΔCt method and using for all genes Actin beta (ACTB) expression as ref. ^55^. The 2−ΔΔCt results were expressed as fold variation relative to the value of undifferentiated cells control for each condition^14^.

### Gene expression analysis

TGF-β, VEGF-A and FGF2 genes expression were evaluated before osteogenic, adipogenic and chondrogenic induction. ALPL, BGLAP, COL1A1, OPG and RUNX2 genes were evaluated for osteogenic induction, ADIPOQ and PPRG genes for adipogenic induction, and ACAN and SOX9 genes for chondrogenic induction. cDNA of cells in osteogenic differentiation was also analyzed for the expression profile of HOX and TALE gene. In particular, 11 genes were evaluated: HOXA1, HOXA6, HOXA7, HOXA11, HOXB8, HOXB9, HOXD9, HOXD10, MEIS2, MEIS3 and PBX1. All these genes were analyzed by specific QuantiTect Primer Assay (Qiagen, Hilden, Germany)^14^. All primers are listed in Table 2.

**Table 2:** Primers qPCR of gene expression analysis.

Symbol	Gene	Primer Fw (5′→3′)		Primer Rv (5′→3′)	T annealing
ACTB*	Actin, beta	CCTTGCACATGCCGGAG		ACAGAGCCTCGCCTTTG	60 °C 20″
HOXA1**	Homeobox A1	Hs_HOXA1_1_SG			55 °C 20″
HOXA6**	Homeobox A6	Hs_HOXA6_1_SG			55 °C 20″
HOXA7**	Homeobox A7	Hs_HOXA7_2_SG			55 °C 20″
HOXA11**	Homeobox A11	Hs_HOXA11_2_SG			55 °C 20″
HOXB8**	Homeobox B8	Hs_HOXB8_2_SG			55 °C 20″
HOXB9**	Homeobox B9	Hs_HOXB9_1_SG			55 °C 20″
HOXD9**	Homeobox D9	Hs_HOXD9_1_SG			55 °C 20″
HOXD10**	Homeobox D10	Hs_HOXD10_1_SG			55 °C 20″
MEIS2**	Meis homebox 2	Hs_MEIS2_1_SG			55 °C 20″
MEIS3**	Meis homebox 3	Hs_MEIS3_1_SG			55 °C 20″
PBX1**	Pre-B-cell leukemia homebox 1	Hs_PBX1_1_SG			55 °C 20″
ACAN***	Aggrecan	TCGAGGACAGCGAGGCC	TCGAGGGTGTAGCGTGTAGAGA		60 °C 20″
ADIPOQ**	Adiponectin	Hs_ADIPOQ_1_SG			55 °C 20″
ALPL**	Alkaline phosphatase liver/bone/kidney isozyme	Hs_ALPL_1_SG			55 °C 20″
BGLAP**	Osteocalcin	Hs_BGLAP_1_SG			55 °C 20″
COL1A1**	Collagen I, chain α1	Hs_COL1A1_1_SG			55 °C 20″
PPARG**	Peroxisome Proliferator-Activated Receptor Gamma	Hs_PPARG_1_SG			55 °C 20″
RUNX2**	Runt related transcription factor 2	Hs_RUNX2_1_SG			55 °C 20″
SOX9**	SRY-box containing gene 9	Hs_SOX9_1_SG			55 °C 20″
TNFRSF11B**	Osteoprotegerin (OPG)	Hs_TNFRSF11B_1_SG			55 °C 20″
FGF2**	Fibroblast growth factor 2	Hs_FGF2_1_SG			55 °C 20″
TGFβ1**	Transforming growth factor-β1	Hs_TGFB1_1_SG			55 °C 20″
VEGFA**	Vascular endothelial growth factor A	Hs_VEGFA_6_SG			55 °C 20″

*Prime Time assay (Integrated DNA Technologies IDT, Coralville, Iowa, USA).

**QuantiTect Primer Assay (Qiagen).

***Designed with Primer Blast (http://www.ncbi.nlm.nih.gov/tools/primer-blast/).

### Statistical analysis

Statistical analysis was performed using R v.3.5.3 software [R: The R Project for Statistical Computing, R Foundation for Statistical Computing. (2008). https://www.r-project.org/ (accessed August 15, 2018)]; package ‘WRS2’^56^ was used to analyse data. Data are reported as mean ± standard deviation (SD) at a significant level of *p*  < 0.05. Cell viability for each experimental time and CFUs data were analyzed with a one-way ANOVA followed by Scheffé post hoc test. After having verified normal distribution (Shapiro-Wilk test) and homogeneity of variance (Levene test), gene expression data were analyzed with robust one-way (*F*_*M*_) and a post hoc comparison test based on trimmed means was used to estimate the effects or the interactions of factors between groups ($\hat{\psi }$ pairwise trimmed mean differences) through percentile bootstrap^57,58^.

## Data Availability

The data reported in this study are available from the corresponding author.

## References

[1] Bain BJ (2003). Bone marrow biopsy morbidity and mortality. Br. J. Haematol..

[2] Kitchel SH, Wang MY, Lauryssen CL (2005). Techniques for aspirating bone marrow for use in spinal surgery. Neurosurg..

[3] Imam MA (2017). A systematic review of the clinical applications and complications of bone marrow aspirate concentrate in management of bone defects and nonunions. Int. Orthop..

[4] Hernigou P, Poignard A, Manicom O, Mathieu G, Rouard H (2005). The use of percutaneous autologous bone marrow transplantation in nonunion and avascular necrosis of bone. J. Bone Jt. Surg. Br..

[5] Hernigou P (2017). Allografts supercharged with bone-marrow-derived mesenchymal stem cells possess equivalent osteogenic capacity to that of autograft: a study with long-term follow-ups of human biopsies. Int. Orthop..

[6] Salamanna F (2017). Mesenchymal Stem Cells for the Treatment of Spinal Arthrodesis: From Preclinical Research to Clinical Scenario. Stem Cell Int..

[7] Schottel PC, Warner SJ (2017). Role of Bone Marrow Aspirate in Orthopedic Trauma. Orthop. Clin. North. Am..

[8] Vaz K (2010). Bone grafting options for lumbar spine surgery: a review examining clinical efficacy and complications. SAS J..

[9] Pesenti S (2017). Bone substitutes in adolescent idiopathic scoliosis surgery using sublaminar bands: is it useful? A case-control study. Int. Orthop..

[10] Riley RS (2004). A pathologist’s perspective on bone marrow aspiration and biopsy: I. Performing a bone marrow examination. J. Clin. Lab. Anal..

[11] Konda B (2014). Safe and successful bone marrow biopsy: an anatomical and CT-based cadaver study. Am. J. Hematol..

[12] Barbanti Brodano G (2013). Mesenchymal stem cells derived from vertebrae (vMSCs) show best biological properties. Eur. Spine J..

[13] Badrinath R, Bohl DD, Hustedt JW, Webb ML, Grauer JN (2014). Only prolonged time from abstraction found to affect viable nucleated cell concentrations in vertebral body bone marrow aspirate. Spine J..

[14] Salamanna F (2018). Biological Rationale for the Use of Vertebral Whole Bone Marrow in Spinal Surgery. Spine ..

[15] Salamanna F (2018). Bone marrow aspirate clot: A technical complication or a smart approach for musculoskeletal tissue regeneration?. J. Cell Physiol..

[16] Lim ZXH (2019). Autologous bone marrow clot as an alternative to autograft for bone defect healing. Bone Jt. Res..

[17] Muschler GF (2003). Spine fusion using cell matrix composites enriched in bone marrow-derived cells. Clin. Orthop. Relat. Res..

[18] Palta S, Saroa R, Palta A (2014). Overview of the coagulation system. Indian. J. Anaesth..

[19] Kim YJ, Bridwell KH, Lenke LG, Rhim S, Cheh G (2006). Pseudarthrosis in long adult spinal deformity instrumentation and fusion to the sacrum: prevalence and risk factor analysis of 144 cases. Spine.

[20] Barbanti Bròdano G (2014). Hydroxyapatite-Based Biomaterials Versus Autologous Bone Graft in Spinal Fusion: An *In Vivo* Animal Study. Spine.

[21] Schroeder J, Kueper J, Leon K, Liebergall M (2015). Stem cells for spine surgery. World J. Stem Cell.

[22] Tural-Kara T, Özdemir H, Fitöz S, Çiftçi E, Yalçınkaya F (2016). Bone marrow aspiration complications: Iliopsoas abscess and sacroiliac osteomyelitis. Turk. J. Pediatr..

[23] Horn P (2008). Isolation of human mesenchymal stromal cells is more efficient by red blood cell lysis. Cytotherapy.

[24] Italiano JE (2008). Angiogenesis is regulated by a novel mechanism: pro- and antiangiogenic proteins are organized into separate platelet alpha granules and differentially released. Blood.

[25] Schallmoser K (2007). Human platelet lysate can replace fetal bovine serum for clinical-scale expansion of functional mesenchymal stromal cells. Transfus..

[26] Grafe I, Alexander S, Peterson JR (2018). TGF-beta family signaling in mesenchymal differentiation. Cold Spring Harb. Perspect. Biol..

[27] Ferrara N, Gerber HP (2001). The role of vascular endothelial growth factor in angiogenesis. Acta Haematol..

[28] Tsutsumi A (2001). Retention of multilineage differentiation potential of mesenchymal cells during proliferation in response to FGF. Biochem. Biophys. Res. Commun..

[29] Dominici M (2006). Minimal criteria for defining multipotent mesenchymal stromal cells. The International Society for Cellular Therapy position statement. Cytotherapy.

[30] Reinisch A (2015). Epigenetic and *in vivo* comparison of diverse MSC sources reveals an endochondral signature for human hematopoietic niche formation. Blood.

[31] Yoshimura K (2006). Characterization of freshly isolated and cultured cells derived from the fatty and fluid portions of liposuction aspirates. J. Cell Physiol..

[32] Ferraro GA (2013). Human adipose CD34+ CD90+ stem cells and collagen scaffold constructs grafted *in vivo* fabricate loose connective and adipose tissues. J. Cell Biochem..

[33] Sidney LE, Branch MJ, Dunphy SE, Dua HS, Hopkinson A (2014). Concise review: evidence for CD34 as a common marker for diverse progenitors. Stem Cell.

[34] Quirici N (2002). Isolation of bone marrow mesenchymal stem cells by anti-nerve growth factor receptor antibodies. Exp. Hematol..

[35] Kuçi S (2010). CD271 antigen defines a subset of multipotent stromal cells with immunosuppressive and lymphohematopoietic engraftment-promoting properties. Haematologica.

[36] Simmons PJ, Torok-Storb B (1991). CD34 expression by stromal precursors in normal human adult bone marrow. Blood.

[37] Kopher RA (2010). Human embryonic stem cell-derived CD34+ cells function as MSC progenitor cells. Bone.

[38] Woodfin A, Voisin MB, Nourshargh S (2007). PECAM-1: a multi-functional molecule in inflammation and vascular biology. Arterioscler. Thromb. Vasc. Biol..

[39] Gheisari Y, Ahmadbeigi N (2016). Mesenchymal Stem Cells and Endothelial Cells: A Common Ancestor?. Arch. Iran. Med..

[40] De Girolamo L (2013). Mesenchymal stem/stromal cells: a new “cells as drugs” paradigm. Efficacy and critical aspects in cell therapy. Curr. Pharm. Des..

[41] Schieker M (2004). The use of four-colour immunofluorescence techniques to identify mesenchymal stem cells. J. Anat..

[42] Zhu H (2006). The role of the hyaluronan receptor CD44 in mesenchymal stem cell migration in the extracellular matrix. Stem Cell.

[43] Suto EG (2017). Prospectively isolated mesenchymal stem/stromal cells are enriched in the CD73+ population and exhibit efficacy after transplantation. Sci. Rep..

[44] Rege TA, Hagood JS (2006). Thy-1, a versatile modulator of signaling affecting cellular adhesion, proliferation, survival, and cytokine/growth factor responses. Biochim. Biophys. Acta.

[45] Caplan AI (2005). Review: mesenchymal stem cells: cell-based reconstructive therapy in orthopedics. Tissue Eng..

[46] Bielby R, Jones E, McGonagle D (2007). The role of mesenchymal stem cells in maintenance and repair of bone. Injury.

[47] Hanna H, Mir LM, Andre FM (2018). *In vitro* osteoblastic differentiation of mesenchymal stem cells generates cell layers with distinct properties. Stem Cell Res. Ther..

[48] Wright EM, Snopek B, Koopman P (1993). Seven new members of the Sox gene family expressed during mouse development. Nucleic Acids Res..

[49] Rux DR, Wellik DM (2017). Hox genes in the adult skeleton: Novel functions beyond embryonic development. Dev. Dyn..

[50] Gordon JA (2010). Pbx1 represses osteoblastogenesis by blocking Hoxa10-mediated recruitment of chromatin remodeling factors. Mol. Cell. Biol..

[51] Roson-Burgo B, Sanchez-Guijo F, Del Cañizo C, De Las Rivas J (2014). Transcriptomic portrait of human Mesenchymal Stromal/Stem Cells isolated from bone marrow and placenta. BMC Genomics.

[52] Piek E (2010). Osteo-transcriptomics of human mesenchymal stem cells: accelerated gene expression and osteoblast differentiation induced by vitamin D reveals c-MYC as an enhancer of BMP2-induced osteogenesis. Bone.

[53] Saulnier N (2011). Gene profiling of bone marrow- and adipose tissue-derived stromal cells: a key role of Kruppel-like factor 4 in cell fate regulation. Cytotherapy.

[54] Davidson AJ (2003). cdx4 mutants fail to specify blood progenitors and can be rescued by multiple hox genes. Nat..

[55] Livak KJ, Schmittgen TD (2001). Analysis of relative gen e expression data using real-time quantitative PCR and the 2(-Delta Delta C(T))Method. Methods.

[56] Mair P., Wilcox R. Robust Statistical Methods Using WRS2 package. Behav Res Methods (2019). 10.3758/s13428-019-01246-w 31152384

[57] Wilcox RR, Tian ST (2011). Measuring effect size: a robust heteroscedastic approach for two or more groups. J. Appl. Stat..

[58] Wilcox Rand (2017). Robust Regression. Introduction to Robust Estimation and Hypothesis Testing.

